# Supplementary material for: Heavy Metal Transporters-Associated Proteins in Solanum tuberosum: Genome-Wide Identification, Comprehensive Gene Feature, Evolution and Expression Analysis
Source: Genes (Basel). 2020 Oct 28;11(11):1269. doi: 10.3390/genes11111269 (PMC7694169; doi:10.3390/genes11111269)
Supplement: Supplementary file 1 [file genes-11-01269-s001.pdf]

SUPPLEMENTARY MATERIALS

Heavy Metal Transporters-Associated Proteins in *S. tuberosum*： Genome-wide Identification, Comprehensive Gene Feature, Evolution and Expression Analysis

Guandi He [1], Lijun Qin [1], Weijun Tian [2], Lulu Meng [2], Tengbing He [2,3,*] and Degang Zhao [1,4,*]

[1] The Key Laboratory of Plant Resources Conservation and Germplasm Innovation in Mountainous Region (Ministry of Education), Institute of Agro-Bioengineering and College of Life Sciences, Guizhou University, Guiyang 550025, China; gs.gthe15@gzu.edu.cn (G.H.); ljqin@gzu.edu.cn (L.Q.)
[2] Agricultural College, Guizhou University, Guiyang 550025, China; gs.tianwj18@gzu.edu.cn (W.T.); gs.llmeng18@gzu.edu.cn (L.M.)
[3] Institute of New Rural Development of Guizhou University, Guiyang 550025, China
[4] Guizhou Academy of Agricultural Science, Guiyang 550025, China
* Correspondence: tbhe@gzu.edu.cn (T.H.); dgzhao@gzu.edu.cn (D.Z.)

Supplementary table legends

Table S1: qRT-PCR primers for expression analysis of *StHMA* gene family in *Solanum tuberosum*;
Table S2: The IDs of 36 HMA genes as well as their properties in *S. tuberosum*;
Table S3: Subcellular localization prediction of StHMA gene family in potato;
Table S4: Ka/Ks ratios of homologous gene pairs between potato and different species;
Table S5: Motif sequences of heavy-metal associated proteins in *S. tuberosum*, *A. thaliana*, *O. sativa* and *S. lycopersicum*;
Table S6: Abiotic stress-related cis-acting elements in the promoter region of StHMA genes;
Table S7: RNA-seq data of potato *StHMA* genes in different tissues.

Table S1. qRT-PCR primers for expression analysis of *StHMA* gene family in *Solanum tuberosum.*

Gene	Transcripts ID	Primer name	Primer sequence
EF-1α	-	*EF1a-F*	TCCTTACCTGAACGCCTGTCA
		EF1a-R	ATTGGAAACGGATATGCTCCA
StHMA1	*PGSC0003DMT400043033*	*StHMA1-F*	CCCATTTTGCTCACCCTTCC
		StHMA1-R	CCGGTTTATGCGACTCACAG
StHMA2	*PGSC0003DMT400045359*	*StHMA2-F*	ACATCTGATTCGCTGCTTGG
		StHMA2-R	ACATGGTGTGAGATTTGGCG
StHMA4	*PGSC0003DMT400009920*	*StHMA4-F*	GCCCTCCTTGTTGCATACTG
		StHMA4-R	AACAGGGGAAAGGAGGGAAC
StHMA8	*PGSC0003DMT400069249*	*StHMA8-F*	GCCTTCTTACCTGTGCGATG
		StHMA8-R	CTGTGAAGGCTGTGAGAGGA
StHMA11	*PGSC0003DMT400018392*	*StHMA11-F*	ACACTCTCGCTGGATCAACA
		StHMA11-R	CGACGTACCAAAAGGAGACC
StHMA16	*PGSC0003DMT400016647*	*StHMA16-F*	CACCTGAAGTTTGGAGTCCG
		StHMA16-R	GGCTCACCTCGATGTTTAGTG
StHMA17	*PGSC0003DMT400029503*	*StHMA17-F*	TGTTTCCTAGGTCCGTCGTT
		StHMA17-R	CCTTTGCCAAAACCACCAGA
StHMA19	*PGSC0003DMT400060398*	*StHMA19-F*	CAGTGGATTGTGCTGCCTTC
		StHMA19-R	ATGGACTGTGAAGGTTGTGC
StHMA22	*PGSC0003DMT400001032*	*StHMA22-F*	ACCCAACACTAACAGTACCCT
		StHMA22-R	TCTATGTCAGTTTGCACGCAG
StHMA25	*PGSC0003DMT400021739*	*StHMA25-F*	GGTTCTGCATTCTCCACACC
		StHMA25-R	CTCCAATCCCAAAACCACCG
StHMA31	*PGSC0003DMT400024481*	*StHMA31-F*	CGTCACAATGCTGCTGAACT
		StHMA31-R	ACTAAGAAGAAGGTGGCGCT
StHMA33	*PGSC0003DMT400015784*	*StHMA33-F*	CACGGACTTTGTTTCGGGTT
		StHMA33-R	TGGGCAAAATGGAAGGTGTT
StHMA34	*PGSC0003DMT400083194*	*StHMA34-F*	CTCCTTTTCCTTTGCCGTCC
		StHMA34-R	AGAAAGTTACGTGCGATGCC
StHMA35	*PGSC0003DMT400040203*	*StHMA35-F*	AGGACGAAGAAGCTGTTGGA
		StHMA35-R	ATCAACTCGTTCATGCCCTG
StHMA36	*PGSC0003DMT400073578*	*StHMA36-F*	TGGAAAGCTAGAGTTGGAGGT
		StHMA36-R	TGGCGTTGTAGAAATGAGGAAG

Gene-specific primers for real-time reverse transcription quantitative PCR (RT-qPCR) were designed by the Primer 6. F, forward; R, reverse.

Table S2. The IDs of 36 HMA genes as well as their properties in *S. tuberosum*.

Gene name	Transcripts ID	Positive or negative chains	Amino acid	Molecular weight	Isoelectric point	Formula	Chromosome location
StHMA1	PGSC0003DMT400015954	+	380	43356.31	5.78	$C_{1897}H_{2823}N_{547}O_{605}S_{12}$	3991348-3993998
StHMA2	PGSC0003DMT400029503	+	291	32740.54	6.9	$C_{1434}H_{2220}N_{410}O_{449}S_{11}$	4674833-4676481
StHMA3	PGSC0003DMT400083194	-	565	57889.92	8.58	$C_{2426}H_{3843}N_{741}O_{798}S_{54}$	39130570-39131689
StHMA4	PGSC0003DMT400073578	+	436	46116.6	8.61	$C_{1939}H_{3031}N_{589}O_{632}S_{44}$	255158-259620
StHMA5	PGSC0003DMT400063687	-	372	40439.98	6.81	$C_{1765}H_{2789}N_{491}O_{581}S_{8}$	5119621-5121945
StHMA6	PGSC0003DMT400032792	-	70	7687.09	9.3	$C_{335}H_{554}N_{96}O_{96}S_{7}$	52462897-52464137
StHMA7	PGSC0003DMT400032707	-	234	24180.04	7.64	$C_{1031}H_{1671}N_{313}O_{340}S_{9}$	47318701-47321428
StHMA8	PGSC0003DMT400015784	-	153	17285.76	9.16	$C_{754}H_{1206}N_{222}O_{226}S_{9}$	81897447-81900463
StHMA9	PGSC0003DMT400024409	-	1002	107196.01	5.16	$C_{4819}H_{7704}N_{1246}O_{1428}S_{39}$	49903753-49905385
StHMA10	PGSC0003DMT400009388	-	121	138879.87	9.15	$C_{610}H_{964}N_{178}O_{179}S_{7}$	87577228-87580997
StHMA11	PGSC0003DMT400078635	+	148	16948.78	9.98	$C_{746}H_{1219}N_{221}O_{211}S_{9}$	76432065-76434200
StHMA12	PGSC0003DMT400043033	+	158	17904.9	9.71	$C_{805}H_{1288}N_{220}O_{225}S_{8}$	48216723-48218378
StHMA13	PGSC0003DMT400037204	-	139	15474.69	7	$C_{696}H_{1071}N_{183}O_{203}S_{7}$	77329843-77331651
StHMA14	PGSC0003DMT400068079	+	326	36878.9	6.12	$C_{1612}H_{2603}N_{443}O_{515}S_{14}$	52417462-52421546
StHMA15	PGSC0003DMT400001501	-	304	33894.57	5.11	$C_{1481}H_{2400}N_{398}O_{479}S_{14}$	27194995-27203856
StHMA16	PGSC0003DMT400015350	+	147	16606.14	9.49	$C_{731}H_{1177}N_{201}O_{221}S_{9}$	58029029-58030494
StHMA17	PGSC0003DMT400038828	-	273	31631.03	5.3	$C_{1365}H_{2257}N_{377}O_{452}S_{14}$	46312673-46314684
StHMA18	PGSC0003DMT400056666	+	152	17169.87	9.4	$C_{759}H_{1214}N_{202}O_{228}S_{11}$	61538313-61541184
StHMA19	PGSC0003DMT400024479	+	150	16456.18	9.46	$C_{729}H_{1187}N_{197}O_{216}S_{9}$	42949396-42951085
StHMA20	PGSC0003DMT400024481	+	150	16502.2	9.46	$C_{733}H_{1185}N_{197}O_{216}S_{9}$	55464451-55465720
StHMA21	PGSC0003DMT400078487	-	414	46005.21	8.1	$C_{1952}H_{3070}N_{598}O_{635}S_{29}$	53411225-53413634
StHMA22	PGSC0003DMT400056199	-	154	17393.09	9.45	$C_{767}H_{1231}N_{211}O_{229}S_{10}$	2063369-2064790
StHMA23	PGSC0003DMT400021739	-	363	40443.68	6.66	$C_{1780}H_{2750}N_{488}O_{547}S_{22}$	9201119-9206477
StHMA24	PGSC0003DMT400060398	+	77	8105.27	5.31	$C_{349}H_{576}N_{94}O_{116}S_{5}$	5557033-5561866
StHMA25	PGSC0003DMT400069249	-	336	38003.29	5.91	$C_{1668}H_{2708}N_{462}O_{526}S_{11}$	45208003-45209479
StHMA26	PGSC0003DMT400018392	+	253	28277.42	8.88	$C_{1220}H_{2035}N_{351}O_{392}S_{12}$	41760071-41760891
StHMA27	PGSC0003DMT400079324	+	180	20955.88	9.1	$C_{926}H_{1428}N_{268}O_{266}S_{12}$	55143456-55145580
StHMA28	PGSC0003DMT400009920	+	367	39867.1	8.52	$C_{1734}H_{2798}N_{484}O_{560}S_{15}$	62455552-62456793
StHMA29	PGSC0003DMT400016647	+	221	25708.87	9.23	$C_{1153}H_{1869}N_{309}O_{337}S_{8}$	40987375-40990668
StHMA30	PGSC0003DMT400045359	-	300	32396.97	9.57	$C_{1391}H_{2316}N_{404}O_{453}S_{14}$	62479681-62480683
StHMA31	PGSC0003DMT400072448	+	166	18145.94	9.38	$C_{799}H_{1327}N_{227}O_{244}S_{4}$	5176268-5179031

StHMA32	PGSC0003DMT400034599	-	140	16163.83	6.36	$C_{697}H_{1171}N_{199}O_{217}S_{11}$	47049115-47050456
StHMA33	PGSC0003DMT400050141	+	106	11370.89	4.72	$C_{497}H_{802}N_{128}O_{165}S_{5}$	11452548-11454378
StHMA34	PGSC0003DMT400020973	+	341	36542.76	9.21	$C_{1571}H_{2587}N_{453}O_{505}S_{20}$	81766806-81768151
StHMA35	PGSC0003DMT400040203	+	260	28825.83	9.76	$C_{1251}H_{2027}N_{363}O_{391}S_{13}$	50982683-50987786
StHMA36	PGSC0003DMT400001032	-	149	16708.3	5.78	$C_{732}H_{1183}N_{199}O_{224}S_{11}$	20055033-20059032

Table S3. Subcellular localization prediction of StHMA gene family in potato.

Gene	Cytoplasmic	Nucleus	Peroxisome	Plasma membrane	Mitochondrion	Chloroplast	Extracellular matrix	Vacuole	Endoplasmic reticulum
StHMA1	—	10	2	—	—	1	—	—	—
StHMA2	1	10	—	—	—	2	—	—	—
StHMA3	6	7	—	—	—	—	—	—	—
StHMA4	1	8	2	2	—	—	—	—	—
StHMA5	—	13	—	—	—	—	—	—	—
StHMA6	2	6	—	—	1	4	—	—	—
StHMA7	—	—	—	—	3	11	—	—	—
StHMA8	—	2	—	—	—	11	—	—	—
StHMA9	4	—	—	6	—	4	—	—	1
StHMA10	4	—	—	—	2	7	—	—	—
StHMA11	—	3	—	2	2	4	—	—	—
StHMA12	11	—	—	—	—	1	1	—	—
StHMA13	14	—	—	—	—	—	—	—	—
StHMA14	1	1	—	—	—	11	—	—	—
StHMA15	3	—	—	—	—	8	3	—	—
StHMA16	4	2	—	—	—	5	2	—	—
StHMA17	3	7	—	1	—	—	2	—	—
StHMA18	2	1	—	—	—	10	—	—	—
StHMA19	—	—	—	—	—	14	—	—	—
StHMA20	—	—	—	—	—	14	—	—	—
StHMA21	1	4	—	1	7	—	—	—	—
StHMA22	10	—	—	—	—	30	—	—	—
StHMA23	2	3	—	2	1	2	1	2	—

StHMA24	2	—	—	—	—	11	—	—	—
StHMA25	2	5	—	—	—	7	—	—	—
StHMA26	—	—	—	—	—	—	—	—	—
StHMA27	—	—	—	—	—	13	—	—	—
StHMA28	7	7	—	—	—	—	—	—	—
StHMA29	11	1	—	—	1	—	—	—	—
StHMA30	2	1	—	—	—	3	6	1	—
StHMA31	7	6	—	—	—	—	—	—	—
StHMA32	4	—	—	—	1	7	1	—	—
StHMA33	—	—	—	—	1	11	1	—	—
StHMA34	2	4	—	—	—	7	—	—	—
StHMA35	1	2	—	—	—	10	—	—	—
StHMA36	1	2	—	1	2	6	1	—	—

Table S4. Ka/Ks ratios of homologous gene pairs between potato and different species.

Homologous gene pairs	Ka	Ks	Ka/Ks	P-Value(Fisher)	Length	Divergence-Time	ML-Score
itb12g00660&PGSC0003DMT400056666	0.0810865	4.38347	0.0184982	1.55E-84	441	1	-844.906
itb01g02040&PGSC0003DMT400032707	0.236192	1.08547	0.217594	2.60E-38	675	0.469979	-1478.75
itb01g10210&PGSC0003DMT400038828	0.185293	3.2623	0.0567982	7.47E-108	786	1	-1551.19
itb03g24840&PGSC0003DMT400001501	0.186874	1.80372	0.103605	1.07E-88	867	0.593468	-1759.45
itb06g23970&PGSC0003DMT400024479	0.109118	1.2642	0.0863138	1.91E-41	447	0.376053	-866.649
itb07g13670&PGSC0003DMT400018392	0.684371	0.354983	1.9279	6.34E-07	672	0.583759	-1529.54
itb08g07490&PGSC0003DMT400024409	0.0930473	0.887601	0.10483	4.34E-186	2976	0.287233	-5629.53
itb12g00660&PGSC0003DMT400056199	0.0846441	1.71071	0.0494789	5.20E-50	444	0.469529	-842.093
itb12g04120&PGSC0003DMT400078635	0.132712	3.09671	0.0428556	5.98E-72	438	0.854718	-876.914
itb12g19630&PGSC0003DMT400001032	0.216476	1.88086	0.115095	2.62E-42	381	0.602011	-800.57
itb12g21430&PGSC0003DMT400050141	0.0998264	1.86918	0.0534066	4.94E-28	255	0.547936	-488.251
itb12g25750&PGSC0003DMT400037204	0.158335	1.16841	0.135513	5.51E-31	405	0.442904	-832.823

itb13g21880&PGSC0003DMT400015954	0.604739	1.31886	0.458531	5.70E-28	1059	0.783719	-2571.37
itb13g23030&PGSC0003DMT400083194	0.377839	2.82903	0.133557	0	1461	0.977852	-3308.97
itb14g01030&PGSC0003DMT400078487	0.60093	0.626159	0.959709	0.658806	1050	0.60713	-2526.75
itb14g01060&PGSC0003DMT400015350	0.216279	1.7255	0.125343	2.35E-45	435	0.521599	-910.881
itb14g16600&PGSC0003DMT400040203	0.370787	1.58365	0.234134	3.16E-54	714	0.707399	-1613.22
itb15g00310&PGSC0003DMT400009920	0.228158	1.18822	0.192016	6.78E-56	906	0.468059	-1917.03
itb15g11860&PGSC0003DMT400073578	0.288882	1.64023	0.176123	1.01E-105	1221	0.586876	-2691.65
itb15g13030&PGSC0003DMT400063687	0.57349	0.438458	1.30797	0.0148055	885	0.539738	-2044.25
OIS95687&PGSC0003DMT400078487	0.160064	0.613304	0.260987	6.67E-27	1161	0.24622	-2264.05
OIS97914&PGSC0003DMT400009920	0.468792	1.62175	0.289066	4.23E-40	879	0.801679	-1900.19
OIS97914&PGSC0003DMT400072448	0.41457	0.875985	0.473261	1.20E-08	477	0.531936	-1070.96
OIS98725&PGSC0003DMT400056199	0.0642516	0.454818	0.141269	9.94E-14	453	0.146316	-782.362
OIT01620&PGSC0003DMT400001032	0.134146	0.527214	0.254442	6.08E-11	423	0.219811	-797.128
OIT01983&PGSC0003DMT400045359	0.283793	0.992527	0.28593	6.94E-34	840	0.44399	-1853.19
Os01t0507700-01&PGSC0003DMT400078635	0.29937	3.19711	0.0936376	0	441	1	-1023.74
PGSC0003DMT400001032&AT3G24450	0.292729	3.33597	0.0877493	0	399	0.88323	-873.695
PGSC0003DMT400001501&AT5G24580	0.320564	2.21048	0.14502	3.08E-105	858	0.800826	-1894.9
PGSC0003DMT400009920&AT5G03380	0.478632	2.57844	0.185629	0	963	1	-2290.84
PGSC0003DMT400015350&AT1G22990	0.271817	3.45964	0.0785682	0	432	1	-953.168
PGSC0003DMT400018392&AT5G60800	0.415165	2.87861	0.144224	0	732	0.99983	-1646.35
PGSC0003DMT400032707&AT4G33520	0.514392	2.23315	0.230343	1.11E-72	678	1	-1594.31
PGSC0003DMT400037204&AT3G48970	0.176086	3.50565	0.0502291	0	417	1	-876.451
PGSC0003DMT400040203&AT2G28660	0.673518	0.686989	0.980391	0.851156	645	0.676691	-1575.23
PGSC0003DMT400045359&AT2G37390	0.553018	0.752983	0.734436	0.00409719	645	0.598933	-1577.76
PGSC0003DMT400056199&AT4G39700	0.216057	2.82269	0.076543	0	453	0.772722	-950.172
PGSC0003DMT400056666&AT4G39700	0.141552	4.022	0.0351945	0	450	1	-907.438
PGSC0003DMT400063687&AT5G27690	0.772629	1.55993	0.495298	7.24E-25	939	1	-2269.97

PGSC0003DMT400068079&AT5G50740	0.345197	3.68181	0.0937572	0	828	1	-1806.92
PGSC0003DMT400072448&AT2G36950	1.12019	0.439755	2.54731	7.28E-10	423	0.936167	-1031.82
PGSC0003DMT400073578&AT3G05220	0.702438	1.69614	0.414139	1.25E-43	1212	1	-2910.51
PGSC0003DMT400078487&AT1G23000	0.608026	2.00073	0.303902	5.98E-70	924	0.955702	-2264.95
PGSC0003DMT400078635&AT1G71050	0.252213	3.54554	0.0711352	0	438	1	-977.164
PGSC0003DMT400083194&AT3G06130	0.412553	1.37449	0.30015	1.15E-64	1284	0.696085	-2982.4
PGSC0003DMT400009920&Zm00001d032873_T001	0.99499	1.0101	0.985044	0.837601	966	1	-2393.73
PGSC0003DMT400078487&Zm00001d008515_T001	0.719656	1.84144	0.390811	9.26E-54	1095	1	-2662.52
PGSC0003DMT400063687&Zm00001d007130_T001	0.628382	1.9469	0.322761	5.82E-77	876	1	-2141.15
PGSC0003DMT400032707&Zm00001d052801_T001	0.827484	0.700359	1.18151	0.121641	561	0.794401	-1394.38

Table S5. Motif sequences of heavy-metal associated proteins in *S. tuberosum*, *A. thaliana*, *O. sativa* and *S. lycopersicum*.

Motif	Consensus sequence	E-value	Sites	Width
1	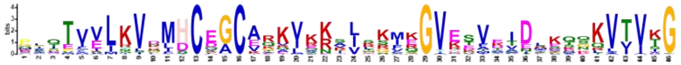<br>Best Possible Match: PLQTVVLKVDMHCEGCARKYKKSLSKMKGVESVEIDLKQQKVTYKG	1.0e-912	50	46
2	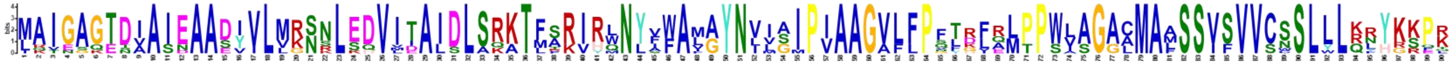<br>Best Possible Match: MAIGAGTDIAIEAADIVLMRSNLEDVITAIDLSRKTFSRIRLNYVWAMAYNVIAIPIAAGVLFPFTRFRLPPWLAGACMAASSVSVVCSSLLLKRYKKPR	1.1e-714	13	100
3	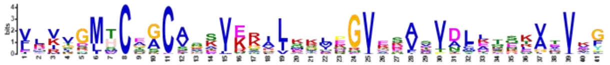<br>Best Possible Match: VLVYGMTCEGCASSYERILKKLPGVFSASYDLLTSKVTVKG	4.5e-605	50	41
4	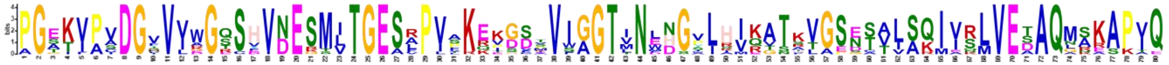<br>Best Possible Match: PGEKVPVDGVVYWGQSHVNESMITGESRPVAKEKGSTVIGGTINLNGVLHIKATKVGSESALSQIVRLVETAQMSKAPYQ	1.8e-542	15	80
5	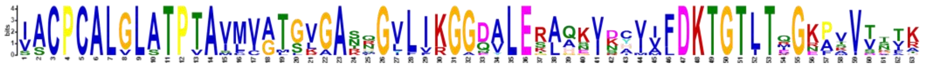<br>Best Possible Match: IACPCALGLATPTAYMVATGVGASQGVLIKGGDALERAQKYDCYIFDKTGTLTQGKPVVTTTK	1.0e-477	15	63
6	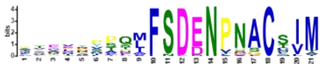<br>Best Possible Match: PTEVYPPQMFSDENPNACSIM	1.2e-358	40	21
7	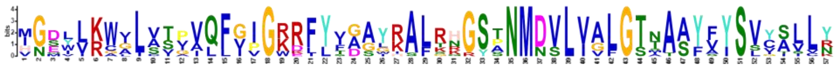<br>Best Possible Match: MGDLLKWYLXTPVQFGIGRRFYYGAYRALRHGSTNMDVLYALGTNAAYFYSVYSLLY	3.6e-319	13	57
8	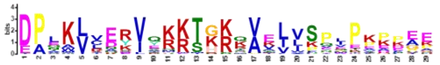<br>Best Possible Match: DPLKLVERYQKKTGKQVELVSPLPKPPEE	1.60E-294	37	29
9	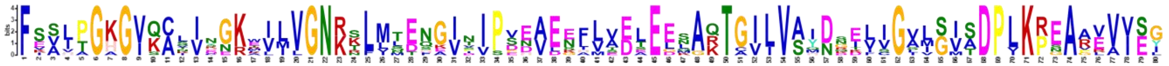	3.50E-229	10	80

	Best Possible Match: FSSLPGKGVQCLINGKWILVGNRSLMTENGINIPVEAEEFLXELEESAQTGILVAIDSELIGVLSISDPLKREAAVVYSG			
10	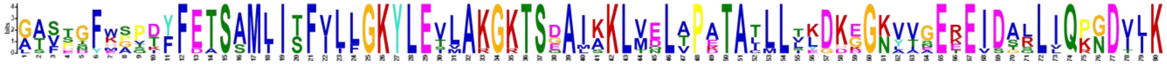	1.30E-298	9	80
	Best Possible Match: GASTGFWSPDYFETSAMLITFYLLGKYLEVLAKGKTSDAIKKLVELAPATATLLTKDKEGKVVGEREIDALLIQPGDYLK			
11	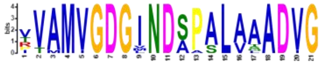	1.30E-164	15	21
	Best Possible Match: VVAMVGDGINDSPALAAADVG			
12	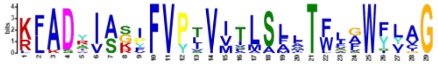	2.20E-144	13	29
	Best Possible Match: KFADYIASIFVPTVITLSLLTFLGWFLAG			
13	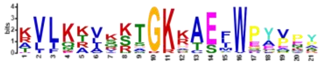	2.00E-139	24	21
	Best Possible Match: KVLKKVKKTGKKAEFWPYVPY			
14	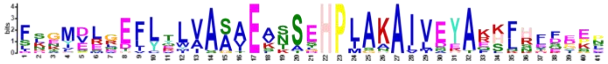	3.50E-172	15	41
	Best Possible Match: FSGMDLGEFLTLVASAEASSEHPLAKAIVEYAKKFHFFEEP			
15	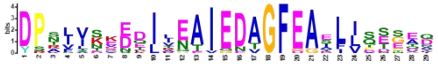	6.70E-148	18	29
	Best Possible Match: DPSLVSKEDILEAIEDAGFEAELISSSEQ			

Table S6. Abiotic stress-related cis-acting elements in the promoter region of StHMA genes.

Gene	ABRE		LTR		MBS	
	Number	Position	Number	Position	Number	Position
StHMA1	3	+703,+704,-1137	0		0	
StHMA2	2	+689,-905	0		2	+224,+253
StHMA3	0		1	+465	0	
StHMA4	1	+798	1	-961	0	
StHMA5	1	-365	2	+1817,+1827	0	
StHMA6	1	-922	0		4	+1649,+960,-1207,-1682
StHMA7	6	+1316,+1319,+1743,+1821,-1318,-1742	1	+407	0	
StHMA8	0		1	-1013	0	
StHMA9	1	+60	1	+1271	0	
StHMA10	5	+1155,+117,+347,-1411,-1413	0		2	-1981,-992
StHMA11	1	-135	2	+143,-292	0	
StHMA12	0		0		0	
StHMA13	2	+753,+754	1	-1412	1	-1058
StHMA14	4	+1758,+1761,-1677,-1760	0		1	-1794
StHMA15	2	+648,+649	0		0	
StHMA16	0		0		0	
StHMA17	0		4	+1260,+1532,+1634,-1186	0	
StHMA18	4	+1100,+1349,-1064,-745	0		0	
StHMA19	6	+590,+591,+627,+629,+630,+653	0		0	
StHMA20	6	+596,+597,+633,+635,+636,+659	0		0	
StHMA21	2	+296,+297	0		0	
StHMA22	3	+179,+181,+182	0		0	
StHMA23	2	+122,+123	0		0	
StHMA24	2	+499,-587	1	+1795	1	+1557
StHMA25	4	+1266,+1806,+1809,-1808	0		1	+1861

StHMA26	2	+139,+1707	0		0	
StHMA27	0		1	+308	0	
StHMA28	1	-1235	1	+301	0	
StHMA29	3	+326,+327,+339	1	-1258	0	
StHMA30	2	+1007,+460	0		1	-738
StHMA31	2	+1234,-1235	0		1	-275
StHMA32	1	-1837	0		0	
StHMA33	1	+1341	0		1	-359
StHMA34	1	+1843	0		0	
StHMA35	2	+213,+214	1	-825	1	-1203
StHMA36	1	+1565	0		0	

**Gene**	**MYB**		**MYC**		**W-box**	
	**Number**	**Position**	**Number**	**Position**	**Number**	**Position**
StHMA1	5	+1821,+437,+652,-1521,-1665	8	+1295,+21,+299,+339,+469,+728,-1531,-1655	1	+1342
StHMA2	5	+1886,+224,+253,+294,-870	4	+1771,+198,+646,+67	0	
StHMA3	1	+1084	1	+1809	0	
StHMA4	1	+1881	2	-153,-710	1	-688
StHMA5	2	-1071,-1807	6	+1048,+1286,+1795,+1914,+547,-290	0	
StHMA6	8	+1045,+1649,+960,-1207,-1499,-1682,-1869,-28	7	+1250,+1373,-1749,-1797,-1851,-236,-409	2	+1234,+1253
StHMA7	3	-1281,-1461,-380	1	+142	0	
StHMA8	13	+1568,+200,+571,+673,-212,-265,-307,-349,-391,-433,-475,-517,-559	5	+1623,+1895,+4,-1164,-1485	0	
StHMA9	3	+1112,+1436,-1148	4	+1753,+665,+966,-1440	1	+1353
StHMA10	3	-1485,-1981,-992	7	+1029,+1218,+1470,+952,-1911,-737,-998	1	+987
StHMA11	2	+1884,+789	3	+909,-1549,-1573	1	+483
StHMA12	1	+1719	3	+1723,-1273,-305	1	-1116
StHMA13	3	+480,-1058,-1783	0		0	

StHMA14	2	-1794,-1858	1	-514	3	+1419,+1470,+79
StHMA15	1	-1706	5	+1172,+1636,+1721,+321,-24	1	-788
StHMA16	2	+1546,-699	4	+171,+482,-259,-30	2	+1578,+987
StHMA17	6	+1326,+1524,+53,-1346,-148,-1488	1	+653	0	
StHMA18	4	+241,+329,+622,-1420	2	+1327,+384	0	
StHMA19	2	+398,-410	0		1	+1073
StHMA20	2	+404,-416	3	+1476,-1144,-24	0	
StHMA21	1	-20	6	+1441,+1688,+1822,+1925,+845,-252	0	
StHMA22	0		2	+1327,+971	0	
StHMA23	4	+1004,+1879,-1930,-711	3	+1051,-1008,-372	1	+1623
StHMA24	1	+1557	1	-1627	1	-1916
StHMA25	5	+1861,+724,-1352,-166,-505	2	+501,+635	0	
StHMA26	4	+1748,+1948,+83,-1475	8	+127,+1514,+322,+869,-238,-287,-30,-575	1	+1311
StHMA27	2	+264,-1281	2	+408,-723	1	-58
StHMA28	1	+1798	4	+1924,+686,+713,-1392	0	
StHMA29	4	+1039,+1459,+1934,-1416	3	+1205,+1467,-351	4	+507,+743,+947,-1413
StHMA30	4	+1574,+1825,+519,-738	6	+1666,+1795,+1879,+1956,-116,-744	0	
StHMA31	5	+1937,+555,-1062,-275,-660	4	+1480,+322,+926,-1043	0	
StHMA32	8	+1216,+272,+422,+47,+83,-1105,-1219,-1851	9	+185,+612,+782,+828,+901,+970,-1202,-1534,-453	0	
StHMA33	2	+466,-359	4	+128,+1659,+571,-985	2	+131,+84
StHMA34	1	-434	5	+919,-1036,-126,-448,-570	1	-1321
StHMA35	1	-1203	6	+462,+626,+928,-293,-419,-542	0	
StHMA36	7	+1327,+1576,+1812,+864,-577,-651,-685	7	+1243,+1294,-1108,-1309,-1350,-1416,-1457	2	+580,-153

Table S7. RNA-seq data of potato *StHMA* genes in different tissues.

	stolon	young tuber	flower	leaf	shoot apex	petiole	stem	mature tuber	root	stamen	tuber peel	tuber cortex	tuber pith	tuber sprout	whole in vitro plant
StHMA1	5.00	4.00	9.00	15.00	1.00	13.00	10.00	3.00	20.00	18.00	0.90	5.00	2.00	9.00	14.00
StHMA2	7.00	3.00	4.00	16.00	0.70	29.00	6.00	3.00	38.00	7.00	1.00	7.00	2.00	4.00	9.00
StHMA3	168.00	163.00	108.00	23.00	104.00	91.00	143.00	90.00	76.00	9.00	67.00	80.00	86.00	105.00	59.00
StHMA4	231.00	192.00	151.00	55.00	48.00	478.00	323.00	57.00	259.00	30.00	237.00	164.00	133.00	725.00	328.00
StHMA5	99.00	34.00	29.00	58.00	15.00	70.00	61.00	16.00	127.00	7.00	7.00	24.00	13.00	57.00	79.00
StHMA6	6.00	1.00	3.00	0.80	0.50	0.00	0.50	0.00	0.00	0.30	0.00	0.00	0.00	0.00	0.00
StHMA7	127.00	72.00	55.00	83.00	140.00	36.00	55.00	80.00	29.00	14.00	54.00	39.00	38.00	62.00	63.00
StHMA8	290.00	299.00	174.00	253.00	230.00	904.00	453.00	173.00	267.00	19.00	162.00	273.00	233.00	309.00	279.00
StHMA9	34.00	34.00	45.00	27.00	25.00	115.00	76.00	48.00	91.00	61.00	53.00	52.00	44.00	66.00	109.00
StHMA10	0.50	0.50	1.00	0.00	0.00	0.50	0.00	2.00	1.00	0.00	4.00	10.00	4.00	29.00	29.00
StHMA11	8.00	16.00	6.00	8.00	13.00	26.00	13.00	10.00	70.00	2.00	7.00	12.00	7.00	93.00	31.00
StHMA12	1.00	8.00	3.00	9.00	2.00	11.00	10.00	0.00	9.00	1.00	1.00	2.00	0.50	8.00	7.00
StHMA13	24.00	4.00	2.00	1.00	2.00	5.00	8.00	0.00	13.00	0.00	0.00	0.00	0.00	4.00	2.00
StHMA14	141.00	42.00	74.00	51.00	39.00	47.00	75.00	89.00	16.00	105.00	73.00	76.00	109.00	34.00	48.00
StHMA15	13.00	156.00	99.00	3.00	80.00	34.00	144.00	194.00	108.00	12.00	104.00	123.00	151.00	40.00	13.00
StHMA16	0.00	0.00	0.00	0.00	0.00	0.00	0.00	0. 00	15.00	0.00	1.00	0.00	0.00	23.00	43.00
StHMA17	114.00	12.00	32.00	24.00	0.00	0.70	8.00	0.00	0.00	0.20	0.00	0.00	0.00	3.00	8.00
StHMA18	8.00	21.00	16.00	2.00	68.00	2.00	32.00	36.00	472.00	0.50	38.00	11.00	11.00	28.00	26.00
StHMA19	0.00	0.50	1.00	0.00	0.00	0.90	0.00	0.00	0.80	0.00	0.00	0.00	0.00	4.00	0.00
StHMA20	0.00	0.70	2.00	0.00	0.00	0.00	0.00	0.00	2.00	0.00	0.00	0.40	0.00	2.00	0.50
StHMA21	198.00	45.00	13.00	32.00	67.00	38.00	36.00	38.00	5.00	0.00	12.00	17.00	24.00	26.00	14.00
StHMA22	0.00	40.00	0.00	0.00	93.00	0.00	0.00	21.00	23.00	0.00	29.00	5.00	2.00	36.00	2.00
StHMA23	0.00	0.50	0.00	0.20	1.00	0.80	0.00	0.30	0.50	1.00	0.70	0.00	0.00	0.50	0.00
StHMA24	59.00	75.00	76.00	60.00	75.00	112.00	65.00	52.00	143.00	87.00	32.00	36.00	23.00	72.00	146.00
StHMA25	52.00	84.00	37.00	16.00	99.00	124.00	179.00	236.00	129.00	22.00	291.00	307.00	497.00	362.00	105.00

StHMA26	7.00	0.00	0.00	0.00	0.60	0.00	0.60	0.00	0.30	0.00	0.00	0.00	0.00	0.00	0.00
StHMA27	1.00	0.00	0.00	0.70	0.90	2.00	0.40	2.00	24.00	0.20	1.00	5.00	2.00	3.00	2.00
StHMA28	30.00	11.00	120.00	30.00	26.00	28.00	71.00	45.00	306.00	318.00	73.00	96.00	133.00	106.00	113.00
StHMA29	0.00	0.00	0.00	0.00	4.00	0.00	0.00	13.00	0.40	0.00	9.00	0.00	0.00	3.00	0.50
StHMA30	19.00	3.00	11.00	18.00	0.40	14.00	6.00	2.00	11.00	3.00	0.90	4.00	2.00	8.00	18.00
StHMA31	3.00	2.00	10.00	5.00	0.50	2.00	0.70	0.30	1.00	13.00	2.00	0.50	0.20	3.00	2.00
StHMA32	2.00	1.00	0.00	6.00	4.00	2.00	3.00	0.60	0.20	0.00	0.60	2.00	1.00	1.00	6.00
StHMA33	59.00	66.00	90.00	68.00	13.00	274.00	311.00	48.00	78.00	150.00	21.00	15.00	16.00	32.00	473.00
StHMA34	35.00	70.00	14.00	15.00	55.00	9.00	15.00	30.00	18.00	2.00	10.00	19.00	15.00	19.00	8.00
StHMA35	7.00	6.00	0.40	4.00	2.00	12.00	5.00	6.00	31.00	0.00	1.00	0.90	0.00	14.00	36.00
StHMA36	5.00	14.00	7.00	3.00	4.00	15.00	24.00	10.00	1.00	2.00	3.00	3.00	2.00	2.00	6.00
